# A Safeguard

**Published:** 1896-01

**Authors:** 


It should be mentined, in the interests of antiseptic purity and suffering humanity, that a good stout tooth-brush, plenty of water, and some antiseptic dentifrice, applied morning and night, afford a greater safeguard against many diseases than many people are aware.—*Sims Woodhead.*

